# Risk Factor–Targeted Perioperative Care Reduces Anastomotic Leakage After Colorectal Surgery: The DoubleCheck Study

**DOI:** 10.1097/SLA.0000000000006442

**Published:** 2024-07-11

**Authors:** Anne de Wit, Boukje T. Bootsma, Daitlin E. Huisman, Bob van Wely, Julie van Hoogstraten, Dirk J.A. Sonneveld, Daan Moes, Johannes A. Wegdam, Carlo V. Feo, Emiel G.G. Verdaasdonk, Walter J.A. Brokelman, David W.G. ten Cate, Tim Lubbers, Emmanuel Lagae, David J.G.H. Roks, Geert Kazemier, Jurre Stens, Gerrit D. Slooter, Freek Daams

**Affiliations:** * Department of Surgery, Amsterdam University Medical Centers, De Boelelaan HV Amsterdam, The Netherlands; † Cancer Center Amsterdam, De Boelelaan HV Amsterdam, The Netherlands; ‡ Department of Surgery, Bernhoven Ziekenhuis, Nistelrodeseweg PT Uden, The Netherlands; § Department of Surgery, Dijklander Ziekenhuis, Maelsonstraat NP Hoorn, The Netherlands; ∥ Department of Surgery, Elkerliek Ziekenhuis, Wesselmanlaan HA Helmond, The Netherlands; ¶ Department of Surgery, Ospedale del Delta, Via Valle Oppio Lagosanto Ferrara, Italy; # Department of Surgery, Jeroen Bosch Ziekenhuis, Henri Dunantstraat GZ ‘s-Hertogenbosch, The Netherlands; ** Department of Surgery, Máxima Medisch Centrum, De Run DB Veldhoven, The Netherlands; †† Department of Surgery, Maastricht University Medical Center, P. Debyelaan, HX, Maastricht, The Netherlands; ‡‡ Department of Surgery, ZorgSaam Ziekenhuis, Wielingenlaan, PA, Terneuzen, The Netherlands; §§ Department of Anesthesiology, Medisch Centrum Leeuwarden, Henri Dunantweg, AD, Leeuwarden, The Netherlands

**Keywords:** anastomotic leakage, colorectal surgery, modifiable risk factors, perioperative care

## Abstract

**Objective:**

The DoubleCheck study aimed to introduce preoperative and perioperative interventions minimizing exposure to modifiable risk factors and determine their effect on colorectal anastomotic leakage (CAL).

**Background:**

CAL is a severe complication. To predict and prevent its occurrence, the LekCheck study identified intraoperative modifiable risk factors for CAL: anemia, hyperglycemia, hypothermia, incorrect timing of antibiotic prophylaxis, administration of vasopressors, and epidural analgesia.

**Methods:**

This international open-labeled interventional study was performed between September 2021 and December 2023. An enhanced care bundle consisting of anemia correction, glucose measurement, attaining normothermia, antibiotics administration within 60 to 15 minutes preoperatively, refraining from vasopressors and epidural analgesia was introduced. The primary outcome was the occurrence of intraoperative risk factors just before the anastomosis creation. Secondary outcomes were CAL and mortality. Univariate and multivariate regression analyses were performed to establish the relationship between the enhanced care bundle, exposure to the 6 factors and CAL.

**Results:**

The historical LekCheck group consisted of 1572 patients versus 902 in the DoubleCheck. The LekCheck group had a mean of 1.84 risk factors versus 1.63 in DoubleCheck (*P*<0.001). In the DoubleCheck, significantly less patients had ≥3 risk factors (*P*<0.001). CAL was significantly lower in the DoubleCheck group (8.6% vs 6.2%, *P*=0.039). The reduction of CAL was associated with the enhanced care bundle in multivariate regression analysis (odds ratio 1.521, 95% CI: 1.01–2.29, *P*=0.045). The mortality rate did not differ significantly (1.3%, vs 0.8%, *P*=0.237).

**Conclusions:**

The DoubleCheck study showed that optimization of modifiable risk factors reduced CAL in colorectal surgery.

Colorectal anastomotic leakage (CAL) is a severe complication after colorectal surgery, with a reported incidence of 3% to 19% worldwide.^1,2^ CAL can have life-altering consequences such as abdominal sepsis, the need for reoperation, a (permanent) stoma, and reduced quality of life, resulting in increased health expenditures.^3^ In oncological patients, CAL is associated with increased local recurrence and reduced survival rates.^4,5^

To predict CAL, research has been targeted on the identification of risk factors.^6^ Among well-known risk factors for CAL are age, history of smoking, higher American Society of Anesthesiologist (ASA) score, anti-cancer therapy, and tumor location.^7–11^ Those factors, however, are difficult or not modifiable. Recently, the emphasis of research has shifted toward the optimization of modifiable risk factors for postoperative complications that are actually adjustable with the introduction of prehabilititation programs and ERAS guidelines.^12,13^

Specifically for CAL, our research group highlighted the importance of modifiable risk factors for its prevention.^6^ A large prospective multicenter study (LekCheck study) identified preoperative anemia (OR 5.40), intraoperative hyperglycemia [odds ratio (OR) 2.80], intraoperative hypothermia (OR 1.39), incorrect timing of antibiotic prophylaxis (OR 1.62), administration of vasopressors (OR 1.80), and epidural analgesia (OR 1.81) among others as modifiable risk factors.^14^ The study showed a correlation between the risk of developing CAL and the number of risk factors present intraoperatively (1.2%–6.4% in patients with 0 to 2 risk factors, respectively, versus 18.7% in patients with ≥3 risk factors present).

Consequently, adequate perioperative management appears to be crucial for improving outcomes of patients undergoing colorectal resections, requiring strict perioperative goals to optimize the intraoperative status of the patient. However, up to now, this is not part of the standard care and no interventional studies have been conducted that have optimized the identified modifiable risk factors for CAL. The aim of the present DoubleCheck study was to optimize the intraoperative condition of the patient by implementation of an intraoperative enhanced care bundle. The secondary aim was to evaluate whether this reduction of intraoperative risk factors led to a decreased CAL-rate as compared with current practice.

## METHODS

### Study Design

Between September 2021 and December 2023, 7 Dutch and 1 Italian hospitals participated in this open-labeled interventional study. An enhanced care bundle was introduced to minimize exposure to the identified modifiable risk factors for CAL and to potentially optimize the preoperative and intraoperative condition of patients undergoing colorectal surgery. The study was approved by the Medical Ethics Review Committee of VU Medical Center and declared exempt from the Medical Research Involving Human Subjects Act. Additional approvals from the Ethics Committee of each individual participating center have been collected and all patients provided written informed consent. The study protocol was registered in the Clinical Trials Registry (ClinicalTrials.gov; NCT05250882).

### Patients

Adult patients who underwent elective colorectal surgery with the creation of a primary anastomosis were eligible for inclusion. Both benign and malignant indications were investigated. Exclusion criteria were the need for emergency surgery, reoperation for complications from recent surgery (within 3 months after the initial procedure), and inability to understand informed consent material.

Historical controls from the previously conducted LekCheck study were used as replacement of a control arm.^14^ During the LekCheck study, the perioperative care was given according to usual practice and was left at the discretion of the local clinicians. The group consisted of 1562 consecutively operated adult patients who underwent colorectal surgery with primary anastomosis from January 2016 to December 2018. The number of patients with zero perioperative risk factors present was 6.7% in this study, with an overall CAL rate of 8.6%.

### Procedures

The enhanced care bundle consisted of interventions to minimize exposure to the modifiable risk factors for CAL that were identified within the LekCheck study (Table 1). Preclinically, hemoglobin, blood glucose, and HbA1c levels were determined. When an iron deficiency anemia was present, it was corrected with intravenous ferric (III) carboxymaltose in the weeks before surgery, and the hemoglobin level was remeasured after infusion.^15^ In case of high blood glucose levels, patients were referred to the internal medicine physician or diabetic nurse for consultation and potential diagnosis and treatment of new-onset diabetes. In addition, blood glucose levels were measured and corrected intraoperatively, and when deviating also postoperatively. During admission, the body temperature was taken tympanically and corrected if necessary with active warming therapy with forced air warming or prewarmed blankets in the holding room. The temperature was measured continuously and corrected when needed intraoperatively. Intravenous antibiotic prophylaxis was administered within 15 to 60 minutes before incision in a single dose (cefazolin and metronidazole).^16,17^ For the use of vasopressive and inotropic drugs, as well as for epidural analgesia, the anesthesia providers team was asked emphatically to use these interventions with clear indications alone.^18^ Consensus was reached among participating colorectal surgeons and anesthesiologists on the interventions in this bundle. At all times, the treating physicians were permitted to amend any of the interventions in both groups to guarantee safe anesthesia and surgery.

**TABLE 1:** The Enhanced DoubleCheck Care Bundle

Modifiable risk factor	Intervention
Anemia	Intravenous ferric (III) carboxymaltose preoperatively
Hyperglycemia	Consultation and potential diagnosis and treatment of new-onset diabetes by internal medicine physician preoperatively
Hypothermia	Active warming therapy preoperatively after hospital admission, on holding, and intraoperatively
Incorrect antibiotic prophylaxis	Correct timing of administration
Vasopressor drug administration	Discouraged in euvolemic state
Epidural analgesia	Discouraged

Additional safety and quality improvement procedures were executed in participating centers, and the ERAS guidelines were completely implemented at the start of the study.^12^ Patients received bowel preparation when undergoing a sigmoid or rectal resection. The introduction of selective bowel decontamination took place during the study period and varied between centers.^17^ The use of indocyanine-green (ICG) was limited to robot-assisted resections. Air testing of the newly created anastomosis was executed on indication (eg, incomplete stapler doughnuts). Intraoperative endoscopy to check the anastomosis was not executed regularly.

Exposure to the modifiable risk factors was prospectively registered by completing an intraoperative web-based checklist during a short time-out procedure just before the creation of the anastomosis in the presence of both the surgical and anesthesiological team. This checklist covered 3 domains: (1) General condition (hemoglobin level, temperature, blood glucose level, moment of antibiotic prophylaxis administration); (2) local perfusion and oxygenation (blood loss, blood transfusion, oxygen saturation, mean arterial pressure, urine production, fluid administration); (3) surgery-related factors (surgery duration, surgical procedure, surgical approach, anastomotic technique, administration of vasopressive agents, epidural analgesia, intraoperative events, additional procedures, contamination of operative field, and stoma placement and type).

Additional baseline characteristics (age, sex, body mass index, ASA-classification, diabetes mellitus, comorbidities, smoking, alcohol, drugs, pathology diagnosis, detection by screening program, tumor distance from anal verge, and neoadjuvant therapy), details about the preoperative course (blood glucose and hemoglobin levels and executed protocol interventions), and postoperative data (laboratory results, length of stay, intensive care unit stay, readmission, CAL, other postoperative complications, reinterventions, and death) were collected prospectively at 30 and 90 days after surgery.

### Outcomes

The primary outcome of the study was the intraoperative condition of the patient, expressed as the number of modifiable risk factors present during the creation of the anastomosis (expressed as mean and proportion ≥3 risk factors). The LekCheck study identified six modifiable risk factors for CAL: anemia, hypothermia, hyperglycemia, incorrect timing of antibiotic prophylaxis, usage of vasopressor drugs, and epidural analgesia.^14^ In the intervention bundle, values were dichotomized according to cutoff levels based on the most recent literature (Table 2).^18–20^ Anemia was defined as a hemoglobin level <7.5 mmol/L (12.1 g/dL) for women and <8.0 mmol/L (12.9 g/dL) for men combined with ferritin <30.0 μg/L or beforementioned hemoglobin levels with ferritin 30.0 to 100.0 μg/L, transferrin saturation <15.0% to 0.0%, and C-reactive protein >5.0 mg/L. Blood glucose levels were considered high when exceeding 10.0 mmol/L (16.1 g/dL) and low under 4.0 mmol/L (6.4 g/dL). A HbA1c level >6.0% or >52.0 mmol/L was defined as hyperglycemia. Hypothermia was defined as a body temperature <36.5 °C. Inadequate timing of the antibiotic prophylaxis was administered outside the window of 60 to 15 minutes before the first incision. Administration of vasopressive or inotropic drugs and epidural analgesia was classified as yes or no.

**TABLE 2:** Modifiable Risk Factors for CAL

Modifiable risk factor	Definition
Anemia	Hemoglobin level ♀<7.5 mmol/L (12.1 g/dL); ♂<8.0 mmol/L (12.9 g/dL)+ferritin<30.0 μg/L or Hemoglobin level ♀<7.5 mmol/L (12.1 g/dL); ♂<8.0 mmol/L (12.9 g/dL)+ferritin 30.0-100.0 μg/L+transferrin saturation<15.0-20.0%+C-reactive protein >5.0 mg/L
Hyperglycemia	Blood glucose level<4.0 mmol/L (6.4 g/dL); >10.0 mmol/L (16.1 g/dL) or HbA1c level >6.0% (52.0 mmol/L)
Hypothermia	Body temperature<36.5 °C
Incorrect antibiotic prophylaxis	Administration<15 min or >60 min before initial incision
Vasopressor drug administration	Yes
Epidural analgesia	Yes

The secondary outcome was 90-day CAL, defined according to the Reisinger definition and categorized according to both the International Study Group of Rectal Cancer (ISREC) and CD classifications.^21–23^ In addition, mortality, length of stay and other postoperative complications were measured.

### Statistical Analysis

Collected data were analyzed using Statistical Package for the Social Sciences software (SPSS 28.0, Chicago, IL). Categorical variables were expressed as proportions (%) and continuous variables as means (SD) or medians (interquartile range), depending on skewness. A sample size calculation was performed and established a number of 853 patients, predicting a clinically relevant increase in the presence of zero modifiable risk factors from 6.7% to 10.0%. The primary study outcome was examined using a Pearson χ^2^-test, Student *t* test, or Mann-Whitney *U* test. Differences between patients with and without CAL were examined using similar descriptive statistics. *P* values <0.05 were considered statistically significant. Logistic regression analysis was performed to analyze the relationship between the intervention protocol, the intraoperative condition defined by exposure to the 6 modifiable risk factors, and the occurrence of CAL. Only patients with a maximum of one missing modifiable risk factor in the intraoperative checklist were considered successful and included in the regression analysis. Afterward, in multivariate analysis, these relationships were corrected for differing baseline and surgery-related factors with *P* values <0.10 and participating hospitals. Results were reported as OR and 95% CI, and *P* values <0.05 were considered statistically significant.

## RESULTS

The intraoperative checklist was executed in 1163 patients at the time of the creation of the anastomosis after the implementation of the enhanced DoubleCheck care bundle. After the removal of patients with >1 missing variable 884 optimized DoubleCheck patients were compared with 1507 historical LekCheck patients. The median age in the total cohort was 69 years (18–95 y), and 52.9% (n=1266) were male. Four hundred eighty-four (20.3%) patients underwent rectal resections and 1873 (79.7%) colonic resections. Executed surgical procedures were 824 (34.5%) right-sided hemicolectomies, 33 (1.4%) extended right-sided hemicolectomies, 17 (0.7%) ileocoecal resections, 48 (2.0%) transverse colonic resections, 242 (10.1% left-sided hemicolectomies, 521 (21.8%) sigmoidectomies, 484 (20.3%) rectal resections, and 221 (9.3%) other resections.

Table 3 displays the patients characteristics in both groups. Groups did not significantly differ for age, sex, or BMI. However, colonic resections were performed more regularly in the DoubleCheck group (77.9% vs 82.2%, *P*=0.012). Compared with the LekCheck patients, more DoubleCheck patients had had an ASA-score ≥3 (ASA-score ≥3: 25.2% vs 29.9%, *P*=0.013). There were less regular smokers in the DoubleCheck group (12.6% vs 9.8%, *P*=0.040).

**TABLE 3:** Study Groups

	LekCheck (n=1507)		DoubleCheck (n=884)		
Variable		Missing		Missing	*P*
Sex (male)	784 (52.0%)		482 (54.5%)		0.237
Age (y)	69 (16)		69 (17)		0.175
Body mass index (kg/m^2^)	26.0 (5)	N=37	26.0 (5)	N=15	0.080
ASA-classification	2 (1)	N=11	2 (1)	N=49	**0.013**
Diabetes mellitus (yes)	225 (15.1%)	N=13	104 (12.2%)	N=31	0.055
Current smoker	180 (12.6%)	N=83	86 (9.8%)	N=8	**0.040**
Alcohol >14 units/wk	101 (7.1%)	N=80	69 (8.6%)	N=81	0.196
Diagnosis (malign%)	1228 (81.8%)	N=6	685 (85.0%)	N=78	0.053
Surgical procedure					**<0.001**
Right-sided hemicolectomy	506 (33.6%)		352 (39.7%)		
Ileocoecal resection	0		17 (1.9%)		
Transversum resection	26 (1.7%)		22 (2.5%)		
Left-sided hemicolectomy	166 (11.0%)		76 (8.6%)		
Sigmoidectomy	288 (19.1%)		233 (26.4%)		
TaTME	0		10 (1.1%)		
TME/LAR	334 (22.2%)		141 (16.0%)		
Other	187 (12.4%)		34 (3.8%)		

Data are presented as numbers (%) or medians (interquartile range).

A *P* value <0.05 was considered statistically significant and marked bold.

CAL indicates colorectal anastomotic leakage.

### Primary Outcome

**FIGURE 1:**
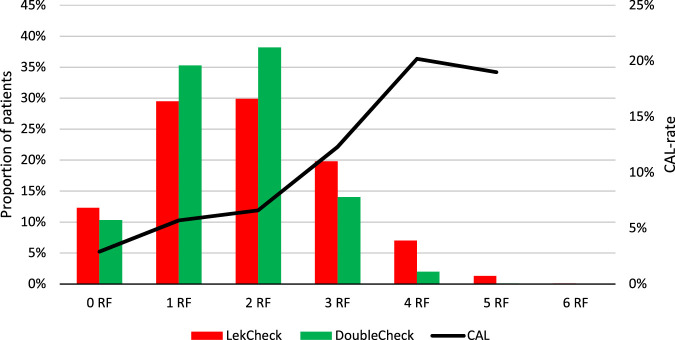
Number of modifiable risk factors related to the occurrence CAL. CAL indicates colorectal anastomotic leakage; RF, risk factors.

The mean number of risk factors in the LekCheck group was 1.84 (0–6) versus 1.63 (0–5) in the DoubleCheck group (*P*<0.001). The proportion of patients with ≥3 modifiable risk factors decreased significantly from 28.3% in the LekCheck group to 16.2% in the DoubleCheck group (*P*<0.001), as displayed in Figure 1. The incidence of epidural analgesia (*P*<0.001) and incorrect antibiotic prophylaxis (*P*<0.001) declined significantly, and no significant effect was observed for alterations in anemia (*P*=0.299), hyperglycemia (*P*=0.077), and hypothermia (*P*=0.780). The administration of vasopressive and inotropic drugs increased (*P*<0.001).

### Secondary Outcome

The CAL-rate in the LekCheck group was 8.6% compared with 6.2% in the optimized DoubleCheck group (*P*=0.039). The mean number of modifiable risk factors present in patients without CAL (n=2207) was 1.72 compared with 2.30 in patients with CAL (n=184) (*P*<0.001). There was no significant difference in the number of days between surgery and diagnosis of CAL between both study groups (5 vs 6 d, *P*=0.168).

Patient and surgical characteristics in both the non-CAL and CAL group are summarized in Table 4. The non-CAL group was 52.2% male, compared with 62.0% in the CAL group (*P*=0.011). Moreover, CAL occurred significantly more often in patients with ASA-score ≥3 (34.4% vs 26.3%, *P*=0.017). The group who received bowel preparation (sigmoid and rectal resections) had a significantly higher CAL-rate compared with the group without bowel preparation (colonic resections) (5.5% vs 10.2%, *P*<0.001). Executed surgical procedures differed significantly between the non-CAL and CAL group (*P*<0.001). CAL patients had significantly longer surgical procedures (169 vs 140 min, *P*<0.001), underwent more open procedures (16.3% vs 9.6%, *P*=0.004), and had more intraoperative blood loss (78 vs 50 mL, *P*<0.001). Creation of a protective stoma was not statistically different between both groups (6.5% vs 9.8%, *P*=0.086); however, creation of a stoma was associated with less severe leaks (ISREC Grade A: 0% vs 0.5%, grade B: 57.1% vs 27.3%, grade C: 42.9% vs 72.1%, *P*=0.009).

**TABLE 4:** Baseline Characteristics

	No CAL (n=2207)		CAL (n=184)		
Variable		Missing		Missing	*P*
Sex (male)	1152 (52.2%)		114 (62.0%)		**0.011**
Age (y)	69 (16)		69 (16)		0.713
Body mass index (kg/m^2^)	26.0 (5)	N=50	26.0 (5)	N=2	0.307
ASA-classification	2 (1)	N=56	2 (1)	N=4	**0.007**
Diabetes mellitus (yes)	296 (13.7%)	N=42	33 (18.0%)	N=1	0.103
Current smoker (yes)	241 (11.4%)	N=85	25 (14.0%)	N=6	0.281
Alcohol >14 units/week (yes)	154 (7.5%)	N=153	16 (9.1%)	N=8	0.445
Drugs (yes)	6 (0.8%)	N=1408	1 (1.9%)	N=131	0.363
Diagnosis (malign)	1761 (82.8%)	N=79	152 (84.9%)	N=5	0.460
Diagnosed by screening program (yes)	672 (34.9%)	N=280	55 (34.0%)	N=22	0.813
Neoadjuvant therapy (yes)	222 (11.1%)	N=203	29 (17.0%)	N=13	0.385
Chemotherapy	49 (2.4%)		3 (1.8%)		
Radiotherapy	110 (5.5%)		17 (9.9%)		
Chemoradiotherapy	63 (3.1%)		9 (5.3%)		
Preoperative SDD (yes)	266 (12.7%)	N=109	24 (13.6%)	N=7	0.736
Bowel preparation (yes)	903 (45.0%)	N=202	103 (61.7%)	N=17	**<0.001**
Surgical procedure					**<0.001**
Right-sided hemicolectomy	814 (36.8%)		43 (23.3%)		
Ileocoecal resection	17 (0.8%)		0		
Transversum resection	45 (2.0%)		3 (1.6%)		
Left-sided hemicolectomy	224 (10.1%)		18 (9.8%)		
Sigmoidectomy	480 (21.7%)		41 (22.3%)		
TaTME	8 (0.4%)		2 (1.1%)		
TME/LAR	415 (18.8%)		60 (32.6%)		
Other	204 (9.3%)		17 (9.2%)		
Surgical approach (open)	211 (9.6%)	N=2	30 (16.3%)		**0.004**
Duration of surgery (minutes)	140 (74%)	N=63	169 (87%)	N=2	**<0.001**
Protective stoma (yes)	143 (6.5%)	N=1	18 (9.8%)		0.086
Additional organ resection (yes)	171 (21.1%)	N=1398	14 (25.5%)	N=129	0.450
Blood loss (mL)	50 (80%)	N=163	77 (50%)	N=14	**<0.001**
Intraoperative blood transfusion (yes)	43 (1.9%)	N=1	4 (2.2%)		0.833
Mean arterial pressure (mm Hg)	81 (16)	N=24	81 (14)	N=1	0.889
Oxygen saturation (%)	98 (2)	N=10	98 (2)		0.258
Fluid administration (mL/h)	426 (307)	N=110	390 (297)	N=4	0.181
Urine production (mL)	165 (200)	N=135	200 (280)	N=5	0.284
Surgeon seniority (gastrointestinal specialized)	1784 (80.8%)	N=1	151 (82.1%)		0.680

Data are presented as numbers (%) or medians (interquartile range).

A *P* value <0.05 was considered statistically significant and marked bold.

CAL indicates colorectal anastomotic leakage; SDD, selective bowel decontamination.

The observed difference in mortality was not statistically significant (1.3% vs 0.8%, *P*=0.237). The mean length of stay was significantly longer in the LekCheck group versus the DoubleCheck group (7.39 vs 5.41 d, *P*<0.001). Other complications did not differ between groups (12.3% vs 13.9%, *P*=0.269) (Table 5).

**TABLE 5:** Postoperative Outcomes

	LekCheck (n=1507), n	DoubleCheck (n=884)	*P*
CAL	129 (8.6%)	55 (6.2%)	**0.039**
ISREC-classification			**<0.001**
A: no intervention	0	1 (1.8)	
B: intervention other than re-operation	57 (38.3%)	5 (9.1%)	
C: re-operation	92 (61.7%)	49 (89.1%)	
Days until diagnosis CAL	5 (4)	6 (6)	0.168
Other complications	186 (12.3%)	123 (13.9%)	0.269
Length of hospital stay (days)	4 (5)	4 (2)	**<0.001**
Length of hospital stay with CAL (days)	19 (19)	16 (12)	0.116
Death	20 (1.3%)	7 (0.8%)	0.237

Data are presented as numbers (%) or medians (interquartile range).

A *P* value <0.05 was considered statistically significant and marked bold.

CAL indicates colorectal anastomotic leakage; ISREC, international study group of rectal cancer.

### Regression Analysis

In univariate regression analysis, the decline in CAL rate was associated with the introduction of the enhanced care bundle (OR 1.411, 95% CI: 1.017–1.957, *P*=0.039). In multivariate regression analysis, this relationship was corrected for significantly differing baseline and surgery-related factors (sex, ASA-score, smoking, bowel preparation, surgical procedure, surgery duration, surgical approach, blood loss, stoma creation, and participating hospital). This analysis revealed a significant association between the introduction of the enhanced DoubleCheck care bundle and the reduction of CAL (OR 1.521, 95% CI: 1.009–2.292, *P*=0.044) (Table 6).

**TABLE 6:** Univariate- and Multivariate Regression Analysis

		Univariate analysis		Multivariate analysis	
	N (%)	OR (95% CI)	*P*	OR (95% CI)	*P*
LekCheck	1507 (63%)	1		1	
DoubleCheck	884 (37%)	1.41 (1.02–1.96)		1.52 (1.01–2.29)	
			**0.039**		**0.045**

Data are presented as numbers (%).

Multivariate analysis was adjusted for sex, ASA-score, smoking, surgical procedure, surgery duration, surgical approach, blood loss, stoma creation, bowel preparation, and participating hospital.

A *P* value <0.05 was considered statistically significant and marked bold.

### Subgroup Analysis

Colonic and rectal anastomoses were separately analyzed and corrected for significantly differing baseline and surgery-related factors in consecutive multivariate regression analyses (Supplement 1, Supplemental Digital Content 1, http://links.lww.com/SLA/F194). For patients with colonic anastomosis, the relationship between the introduction of the enhanced care bundle and CAL was corrected for blood loss, surgical approach, surgery duration, ASA-score, diabetes mellitus, and participating hospital, and revealed a significant relationship (OR 1.709, 95% CI: 1.099–2.658, *P*=0.017). For patients with rectal resections, this relationship was corrected for sex, ASA-score, pathology diagnosis, tumor distance from anal verge, preoperative selective bowel decontamination administration, fluid administration, surgery duration, ICG utilization, and participating hospital, and revealed a nonsignificant relationship (OR 1.426, 95% CI: 0.671–3.031, *P*=0.356).

## DISCUSSION

The study showed a reduction of modifiable risk factors for CAL in patients undergoing colorectal surgery. This reduction led to a decrease in the CAL rate and reduced length of stay. This interventional study is the first to show that prevention of CAL is possible by introducing a targeted bundle of care to optimize modifiable risk factors.

Although the reduction of the mean amount of risk factors present was statistically significant, the absolute reduction (0.19 risk factor) does not reflect the impact on the surgical outcomes. The fact that implementation of the enhanced bundle of care dramatically reduced the number of patients with ≥3 risk factors (who showed an 18.7% CAL rate in the de LekCheck study) contributed to the improved outcomes of the DoubleCheck group. Implementation of the complete enhanced care bundle, rather than optimization of individual risk factors, reduces the risk of developing CAL and should therefore be pursued.

Several previous studies have reported on the importance of optimizing the condition of patients undergoing abdominal surgery and proposed several strategies to reach this aim.^24^ One large randomized controlled trial showed a reduction of overall postoperative complications after implementation of a multimodal prehabilitation program for patients undergoing colorectal cancer surgery, covering 4 domains: high-intensity exercise program, nutritional intervention, psychological support, and smoking cessation.^25^ An additional meta-analysis concluded a preoperative exercise program to be beneficial to prevent postoperative complications after colorectal surgery.^26^ High BMI levels are associated with postoperative complications and could be classified as a modifiable risk factor.^27^ However, the window of opportunity, especially in patients with a malignancy, is limited. Stretching from the moment of diagnosis to surgery, this period might be too short and possibly even counterproductive in terms of prehabilitation objectives. Parallel with our current enhanced care bundle, prehabilitation strategies could lead to optimal surgical preparation. Future research could help to identify the correct personalization of optimization since not all interventions are suitable for frail patients, who might not be able to participate in intensive training programs.

Even though we studied both colonic and rectal resections, minor differences between both procedures should be highlighted. Individual multivariate regression analysis revealed a significant correlation between the intervention bundle and the reduction of CAL in colonic resections, but this association was nonsignificant in rectal resections. This nonsignificant result can mostly be explained by a lower number of inclusions (n=484). Moreover, it has been demonstrated that occurrence of leaks after rectal resections are predominantly multifactorial and caused by fixed variables.^28^ Despite this outcome in the rectum subgroup, we nevertheless recommended to optimize patient conditions to minimize exposure to risk factors. The use of a protective stoma was 22.1% in rectal resections. Next to the observed mitigating effect of a deviation on the severity of the leaks, rectal surgery patients benefitted from the enhanced care bundle and should therefore be optimized.

ICG was utilized in robot-assisted rectal resections during the DoubleCheck study period. Within this small subgroup of our entire study, we could not solidify its protective impact on leakage. We added the usage of ICG as a part of the multivariate regression analysis for the rectal surgery subgroup. We believe that, given the small amount of patients that was treated using ICG, the potential of confounding is negligible.

In this study, there was a significant difference in ISREC classification of the observed CAL. Although this might reflect a difference in the clinical severity of the leak, it should be seen in the light of the grading system itself. The ISREC classification was designed to grade the effect on the therapeutic strategy of the leak and not its clinical impact. A report on the long-term outcome of the patients in this current study could reveal a more detailed insight into the severity of the leaks, when metrics like 1-year stoma-free survival or stoma reversal rates can be delivered.

The choice of an open-labeled intervention study design with historical controls, rather than a randomized controlled trial, was made under the conviction that all patients could potentially benefit from optimization and would not be harmed by any of the interventions, and should therefore not be withheld from receiving enhanced care.

Some limitations of the study are worth mentioning. When comparing LekCheck with DoubleCheck patients, a time bias cannot be ruled out. Patients in both groups were not treated and included simultaneously, and therefore, changes in the CAL rate may have been biased by the factor of time. However, trends in Dutch national audit data do not show a nationwide decrease in the CAL rate in the time frame of the LekCheck and DoubleCheck study.^29^ However, the difference in time did influence the reduction in length of stay, with fast-track discharge becoming more popular over the recent years.^30^ The impact of the contemporary PREHAB study on this outcome parameter has been minimal, since a very limited amount of patients was treated after the nationwide introduction of the prehabilitation protocol.^25^

Another limitation to the study is that other safety procedures to prevent anastomotic leakage (bowel preparation, the indication for a protective stomas, routine air-leak testing, and ICG) were not standardized. Although we assume that their application was equally distributed over both groups, confounding the results of these interventions cannot be excluded. Potential selection bias could be present due to exclusion from the study when reporting >1 missing risk factor. However, between the LekCheck and DoubleCheck groups, patients with and without >1 missing variable did not differ in baseline characteristics, as were available.

Although showing that a decrease in the total number of modifiable risk factors led to reduced leak rates, this study did not reveal the compliance to the study protocol per individual modifiable risk factor. Collecting longitudinal data can potentially nuance feedback on the intraoperative condition. An incorporated feedback system to highlight results and compliance of individual hospitals could provide the practitioners with optimal feedback and further optimization of the intraoperative condition of patients undergoing colorectal surgery.

Strengths of this study include the international multicenter design. In addition, the interventions in our study did not involve expensive or new interventions and are easily available in both general and academic hospitals. Consequently, we believe that its implementation could easily be achieved in elective colorectal surgeries worldwide. Colorectal surgeons and their patients could benefit from further incorporation of the optimization protocol in clinical care pathways and integration of the checklist in the electronic patient files during the preoperative and intraoperative phases. Moreover, individual feedback was provided to the participants on the surgeon and hospital levels during and after the study period, providing not only insight into the study outcomes but also improving individual performance.

In conclusion, CAL was reduced after the introduction of an enhanced care bundle optimizing the intraoperative condition in patients undergoing colorectal surgery. These results call out for paying attention to the individual risk factors of our patients throughout the whole pathway of care. The future challenge consists of ensuring the attentivity of the entire colorectal care team to these factors at all times and the optimization of individual risk factors for CAL before surgery. The DoubleCheck enhanced care protocol could be implemented in clinical practice worldwide to prevent colorectal anastomotic leakage.

## Supplementary Material
