# Supplementary material for: Risk Factor–Targeted Perioperative Care Reduces Anastomotic Leakage After Colorectal Surgery: The DoubleCheck Study
Source: Ann Surg. 2024 Jul 11;283(1):154–61. doi: 10.1097/SLA.0000000000006442 (PMC12695192; doi:10.1097/SLA.0000000000006442)
Supplement: Supplementary file 1 [file sla-283-154-s001.docx]

**Supplements**

**Supplement 1: Subgroup multivariate regression analysis**

|  | **Colon*** |  |  | **Rectum**** |  |  |
| --- | --- | --- | --- | --- | --- | --- |
|  | **No. (%)** | **OR (95% CI)** | **p value** | **No. (%)** | **OR (95% CI)** | **p value** |
| LekCheck | 1174 (62.7%) | 1 |  | 333 (68.8%) | 1 |  |
| DoubleCheck | 699 (37.3%) | 1.709 (1.099-2.658) |  | 151 (31.2%) | 1.426 (0.671-3.031) |  |
|  |  |  | **0.017** |  |  | 0.356 |
| Data is presented as numbers (%).  *Multivariate analysis was adjusted for: blood loss, surgical approach, surgery duration, ASA-score, diabetes mellitus, and participating hospital.  ** Multivariate analysis was adjusted for: sex, ASA-score, pathology diagnosis, tumor distance from anal verge, preoperative SDD administration, fluid administration, surgery duration, ICG utilization, and participating hospital.  A p value <0.05 was considered statistically significant and marked bold. | | | | | | |
